# Supplementary material for: Structural Characterization of the Dimers and Selective Synthesis of the Cyclic Analogues of the Antimicrobial Peptide Cm-p5
Source: Antibiotics (Basel). 2025 Feb 13;14(2):194. doi: 10.3390/antibiotics14020194 (PMC11851992; doi:10.3390/antibiotics14020194)
Supplement: Supplementary file 1 [file antibiotics-14-00194-s001.zip › antibiotics-3428399-supplementary.pdf]

Structural Characterization of the Dimers and Selective Synthesis of the Cyclic Analogues of the Antimicrobial Peptide Cm-p5

Fidel E. Morales-Vicente [1], Luis A. Espinosa [2], Erbio Díaz-Pico [3], Ernesto M. Martell [4], Melaine Gonzalez [4], Gerardo Ojeda [5], Luis Javier González [2], Armando Rodríguez [6,7], Hilda E. Garay [1], Octavio L. Franco [8], Frank Rosenau [9], Anselmo J. Otero-González [4,*] and Ludger Ständker [6,*]

[1] Synthetic Peptide Group, Physics and Chemistry Department, Center for Genetic Engineering and Biotechnology, P.O. Box 6162, 10600 La Habana, Cuba; fidel.morales@cigb.edu.cu (F.E.M.-V.); hilda.garay@cigb.edu.cu (H.E.G.)
[2] Mass Spectrometry Laboratory, Systems Biology Department, Center for Genetic Engineering and Biotechnology, P.O. Box 6162, 10600 La Habana, Cuba; la900415@gmail.com (L.A.E.); luis.javier@cigb.edu.cu (L.J.G.)
[3] Centro de Bioinformática, Simulación y Modelado (CBSM), Facultad de Ingeniería, Universidad de Talca, Talca 3460000, Chile; erbiodiaz68@gmail.com
[4] Center for Protein Studies, Faculty of Biology, University of Havana, 25 Str. and I Str., 10400 La Habana, Cuba; nestmartell@gmail.com (E.M.M.); glezmel93@gmail.com (M.G.)
[5] General Chemistry Department, Faculty of Chemistry, University of Havana, Zapata and G, 10400 La Havana, Cuba; gemojedac@gmail.com
[6] Core Facility for Functional Peptidomics, Ulm University Medical Center, Meyerhofstraße 4, 89081 Ulm, Germany; armando.rodriguez-alfonso@uni-ulm.de
[7] Core Unit of Mass Spectrometry and Proteomics, Ulm University Medical Center, Albert-Einstein-Allee 11, 89081 Ulm, Germany
[8] Centro de Analises Proteomicas e Bioquímicas, Programa de Pos-Graduaçao em Ciencias Genomicas e Biotecnologia, Universidade Catolica de Brasília, Brasília 70790-160, Brazil; ocfranco@gmail.com
[9] Institute of Pharmaceutical Biotechnology, Ulm University, Albert-Einstein Alle 11, 89081 Ulm, Germany; frank.rosenau@uni-ulm.de
* Correspondence: aotero@fbio.uh.cu (A.J.O.-G.); ludger.staendker@uni-ulm.de (L.S.); Tel.: +53-5500-5396 (A.J.O.-G.); +49-(0)731-500-65171 (L.S.)

Table of Contents:

SD1. Differentiation of the cyclic, the parallel and the antiparallel dimers of Cm-p5 by ESI-MS/MS. 2

SD2. Selective synthesis of the cyclic monomer and dimers of CysCysCm-p5. 3

2.1 General protocol for solid-phase peptide synthesis 3

2.2 Selective synthesis of the cyclic monomer of CysCysCm-p5. 4

2.3 Selective synthesis of the parallel dimer of CysCysCm-p5. 7

References 8

Additional Figures and Tables

SD1. Differentiation of the cyclic, the parallel and the antiparallel dimers of Cm-p5 by ESI-MS/MS.

The analytical RP-HPLC profile of the CysCysCm-p5 cyclization shows two main peaks that yields intense multiply charged ions (3+ and 4+) by ESI-MS, that once deconvoluted with MaxEnt1 showed a major signal with an average molecular weight of 2843.46Da (Figure S1 a,b**Error! Reference source not found.**). These signals correspond to the presence of dimers considering two monomers of CysCysCm-p5 linked by two intermolecular disulfide bonds. These dimers correspond to the antiparallel (favored) and parallel dimers that are generated during the cyclization step (Figure S1 c) depending on the sequence orientation of both monomers [1, 2].

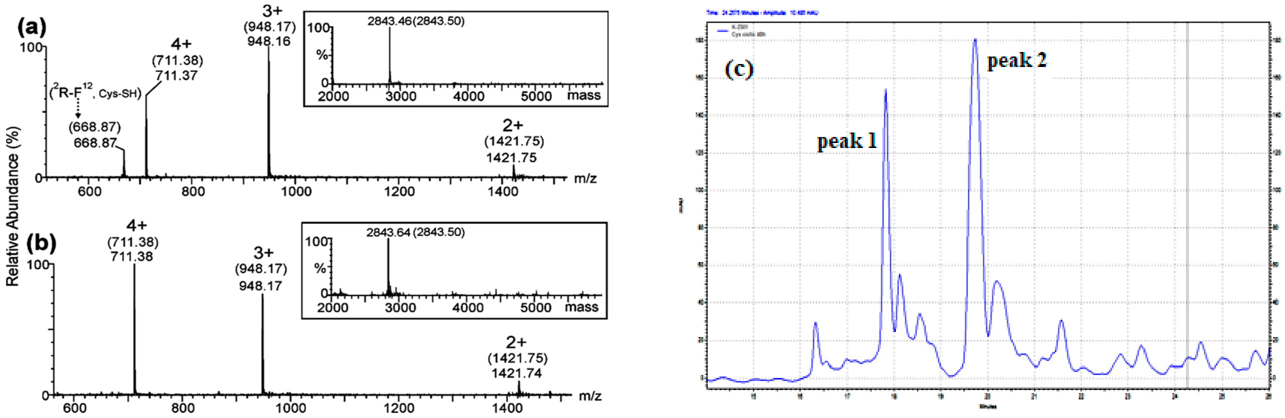

Figure S1: (a) (b) ESI-MS spectra of dimers of CysCysCm-p5. Theoretical monoisotopic species marked in a box and (c) RP-HPLC profiles (Knauer) (oxidation conditions: 0.5mg/ml in water/ACN, pH=8).

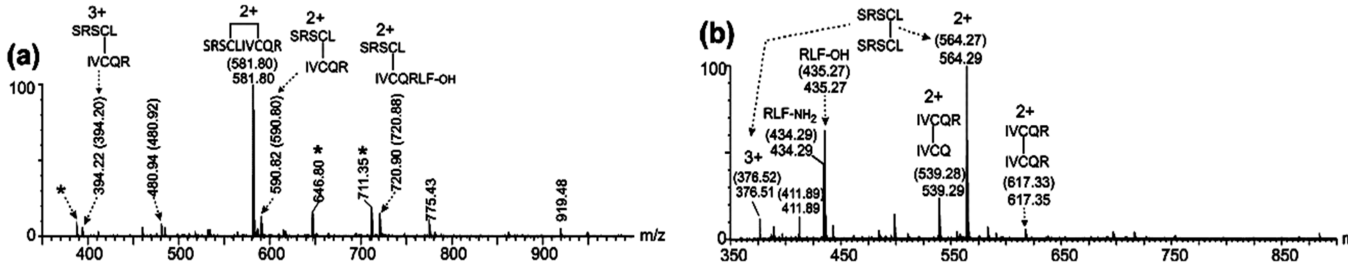

Figure S2: ESI-MS spectra of the chymotrypsin digestion of dimer contained on peak 2 (a) and peak 1 (b) dimers of CysCys-Cm-p5. The expected monoisotopicm/z is indicated in parenthesis.

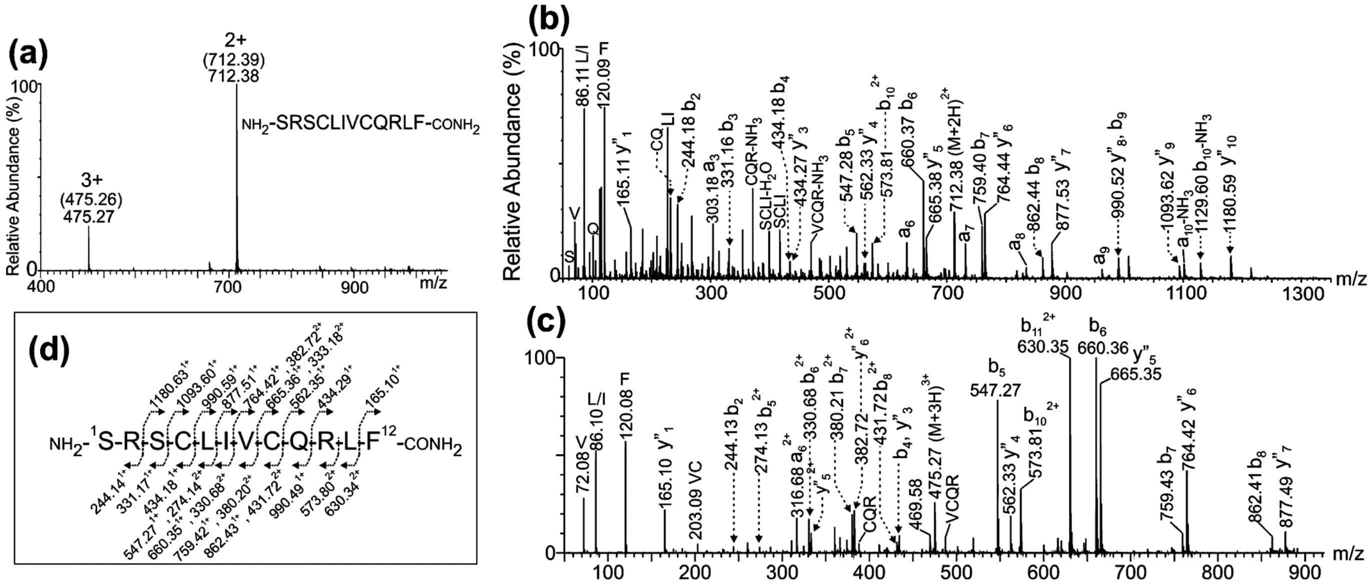

Figure S3: (a) Electrospray ionization mass spectrometry (ESI-MS) analysis of the lineal monomer of Cys-Cm-p5. (b-c) ESI-MS/MS analysis of the $(M+2H)^{2+}$ and $(M+3H)^{3+}$ ions detected at *m/z* 712.38 and 475.27, respectively. (d) Fragmentation scheme of peptide and summary of the assignment for fragment ions observed in the ESI-MS/MS spectra shown in (b) and (c). The expected monoisotopic *m/z* values are indicated in (a) (inside parentheses) and (d).

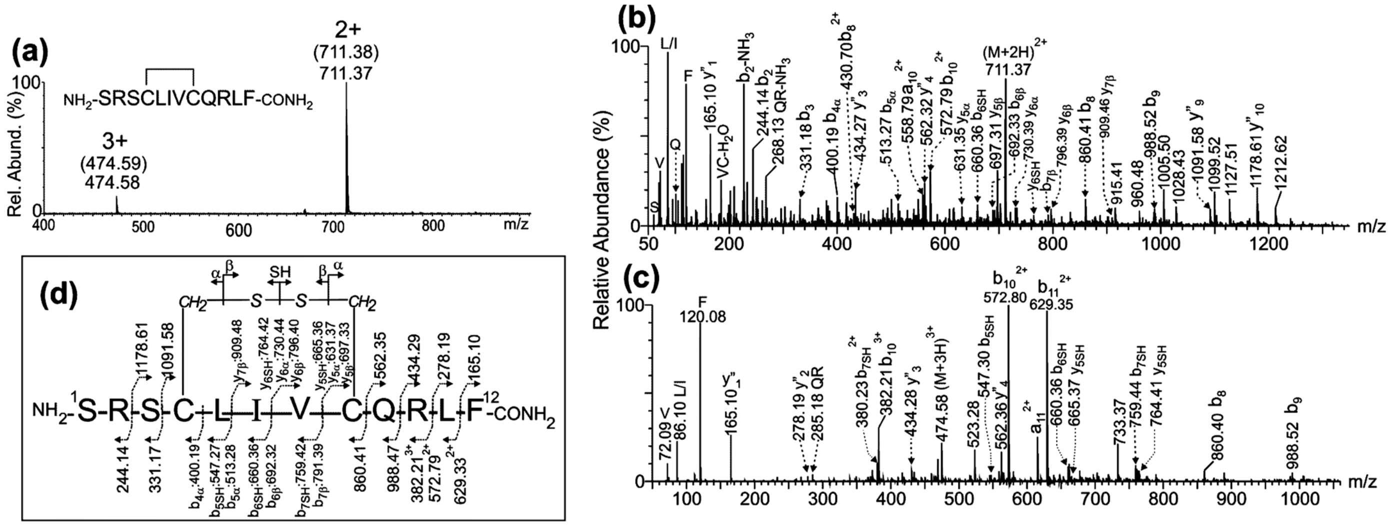

Figure S4: (a) Electrospray ionization mass spectrometry (ESI-MS) analysis of the cyclic monomer of Cys-Cm-p5. (b-c) ESI-MS/MS analysis of the $(M+2H)^{2+}$ and $(M+3H)^{3+}$ ions detected at *m/z* 711.37 and 474.58, respectively. (d) Fragmentation scheme of peptide and summary of the assignment for fragment ions observed in the ESI-MS/MS spectra shown in (b) and (c). The expected monoisotopic *m/z* values are indicated in (a)(inside parentheses) and (d). The nomenclature for the asymmetric fragmentation of the intramolecular disulfide bond (α and β ions) corresponds to a previous paper [3].

The ESI-MS analysis of the lineal (Figure S3) and cyclic (Figure S4) monomer species showed a good agreement between the experimental and expected *m/z* values for the multiply charged ions (2+ and 3+) of CysCysCm-p5 considering the peptide with two free thiols or an intramolecular disulfide bond linking Cys^4-Cys^8, and an amidation at the *C*-terminal. The mass difference of the backbone ions (b_n and y''_n) observed in the MS/MS spectra (Figure S3 b-c) of the $(M+2H)^{2+}$ and $(M+3H)^{3+}$ ions (*m/z* 712.38 and 475.27) confirmed the presence of two free cysteine residues corresponding to the lineal monomer (Figure S3 d) amidated in the *C*-terminal. Figure S4 b-c shows the MS/MS spectra of the precursor ions at *m/z* 711.37 (2+) and 474.58 (3+) and a summary of the cyclic monomer assignment is presented in Figure S4 d. The backbone ions corresponding to y_n (y_1-y_4, y_9-y_{10}) and b_n ions (b_2-b_3, b_8-b_{11}) considering an intramolecular disulfide bond between Cys^4-Cys^8 were detected. A symmetric cleavage of the disulfide bond and further backbone fragmentation generates several signals assigned to y_{5SH}-y_{6SH} and b_{5SH}-b_{7SH} containing Cys^8 and Cys^4 residues as a free thiol group. Additionally, an asymmetric cleavage were detected according to Mormann and coworkers generating α($b_{4\alpha}$, $b_{5\alpha}$, $y_{5\alpha}$, $y_{6\alpha}$) and β($b_{6\beta}$, $b_{7\beta}$, $y_{5\beta}$, $y_{6\beta}$, $y_{7\beta}$) fragment ions with the two Cys residues transformed in dehydroalanine and disulfohydryl cysteine residue, respectively. The nomenclature for the asymmetric fragmentation of the intermolecular disulfide bond (α and β ions) corresponds to a previous paper [3].

SD2. Selective synthesis of the cyclic monomer and dimers of CysCysCm-p5.

2.1 *General protocol for solid-phase peptide synthesis*

Coupling reactions were performed manually on Rink-MBHA resin by a stepwise Fmoc/*t*Bu strategy. Aminoacids were coupled using DIC/Oxyma activation, and completion of the coupling reaction was monitored by the ninhydrin test. Fmoc-deprotection was carried out using 20% piperidine solution in DMF. All final product were cleaved from the resin with the cocktail TFA/TIS/water (95:2.5:2.5), or TFA/TIS/EDT/water (94:1:2.5:2.5) for cysteine containing peptides, precipitated from diethyl ether at -70°C, then taken up in 1:2 ACN/water and lyophilized. All Cm-p5 analogues were analyzed by RP-HPLC, C18 column (Vydac, 4.6 × 150 mm, 5μm) (λ= 226 nm) (linear gradient of 5 to 60 % of solvent B for 35 min, at 0.8mL/min flow) (solvent A: H_2O, 0.1 % TFA; solvent B: CH_3CN, 0.05 % TFA) to determine purity and realize the ESI-MS characterization. In All cases, final products were dissolved in DMSO and purified by preparative RP-HPLC, C18 column (Vydac, 25 × 250 mm, 25μm) (λ= 226 nm) (with a linear gradient of 15 to 80 % of B for 60 min, at 5mL/min flow).

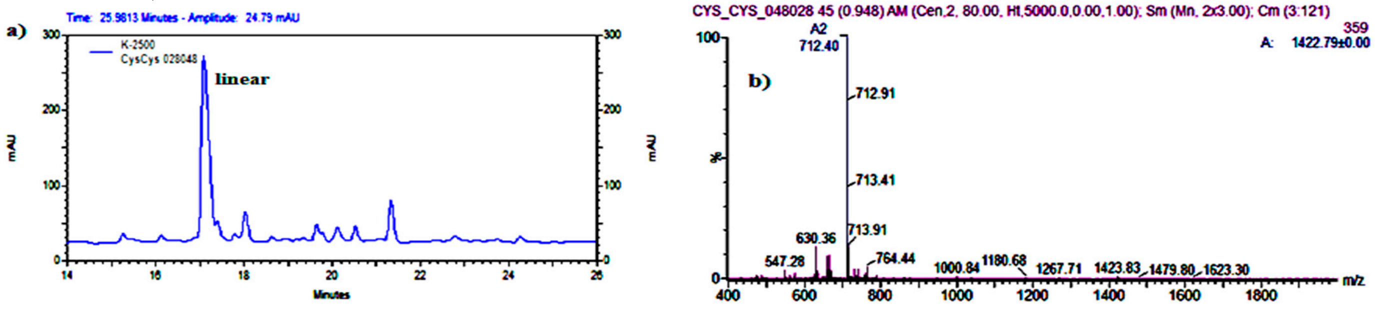

Figure S5: Analytical RP-HPLC profiles (a) (Knauer HPLC system) and ESI-MS (b) of crude CysCysCm-p5 peptide.

2.2 *Selective synthesis of the cyclic monomer of CysCysCm-p5.*

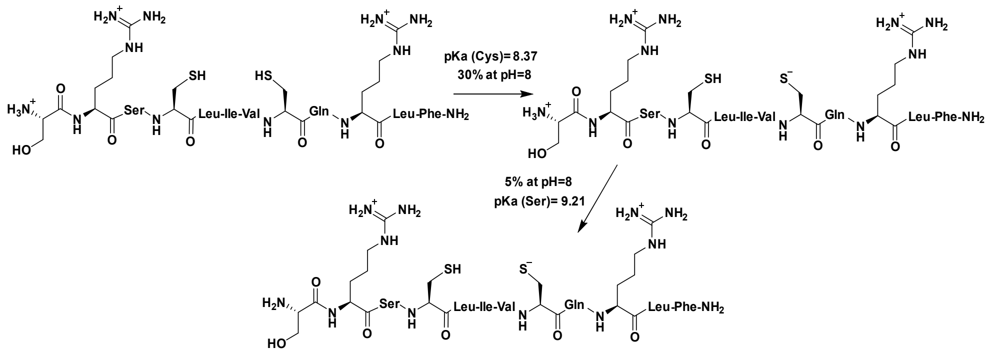

Scheme S1: Ionization of linear CysCysCm-p5 from pH=3 to pH=8.

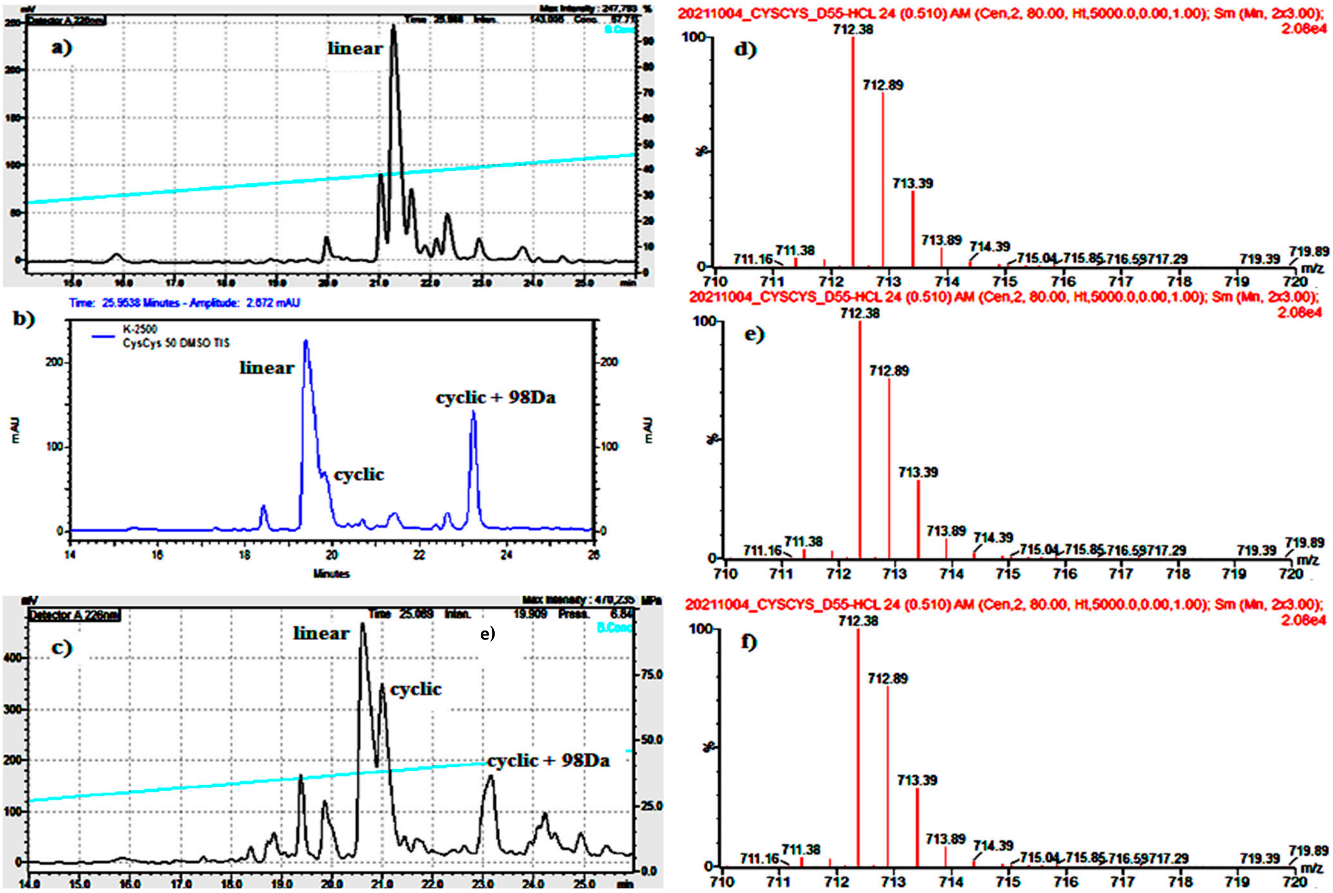

Figure S6: Analytical RP-HPLC profile and ESI-MS of the crude peptide CysCysCm-p5 after the on-resin cyclization with: a, d) DMSO (40%), pH=3, 6h (Chemmatrix 0.41mmol/g, Shimatsu HPLC system), b, e) DMSO 50%, pH=3, 12h (Chemmatrix 0.41mmol/g, Knauer HPLC system) and c, f) DMSO 60%, pH=3, 3h (Rink-MBHA 1.11mmol/g, Shimatsu HPLC system with changed of elution pipeline).

Table S1: Derived compound of Cm-p5 and related molar mass and approximate retention time in RP-HPLC.

Compound	Molar mass	Retention time Knauer	Retention time Knauer new	Retention time Shimatsu	Retention time Shimatsu new
Parallel Dimer	2841.58 g/mol	17.8 min	16.8 min		21.25 min
Linear	1422.79 g/mol	19.3 min	17.4 min	21.25 min	21.6 min
Cyclic	1420.76 g/mol	19.6 min	17.7 min	21.62 min	21.9 min
Cyclic Protected	2037.04 g/mol	-	-	34.2 min	-
Racemic Cyclic	1420.76 g/mol	-	-	-	22.0 min
Antiparallel	2841.56 g/mol	19.8 min	18.2 min	-	23.4 min
Cyclic with adduct	1518.82 g/mol	-	23.25 min	23.8 min	23.8 min

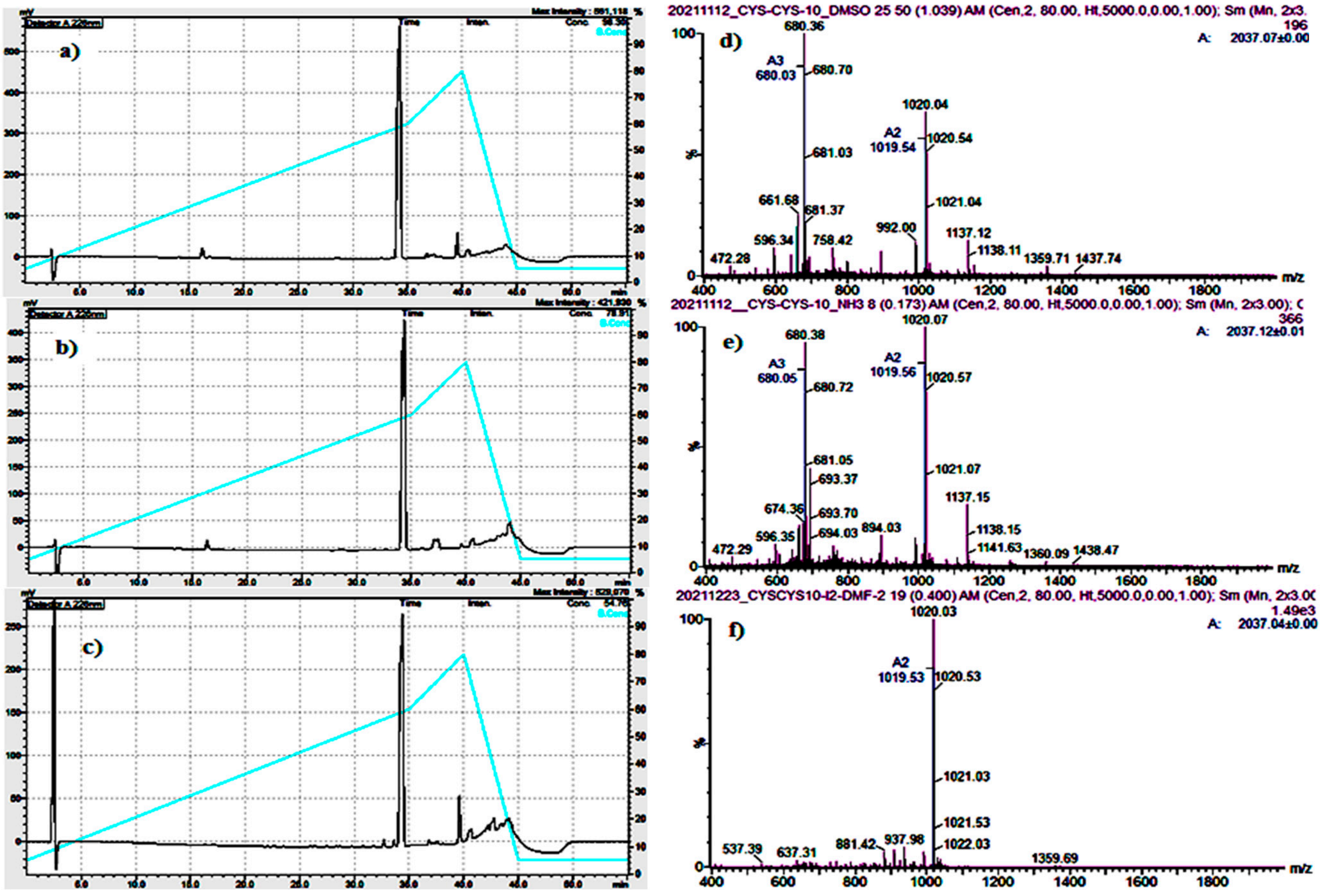

Figure S7: Analytical RP-HPLC (upper panel) profiles and ESI-MS (lower panel) of the protected cyclic peptide CysCysCm-p5 after the on resin (Chemmatrix) cyclization with: a, d) 35% DMSO; b, e) O_2/NH_3 in THF/H_2O (2:1); c, f) I_2/DMF.

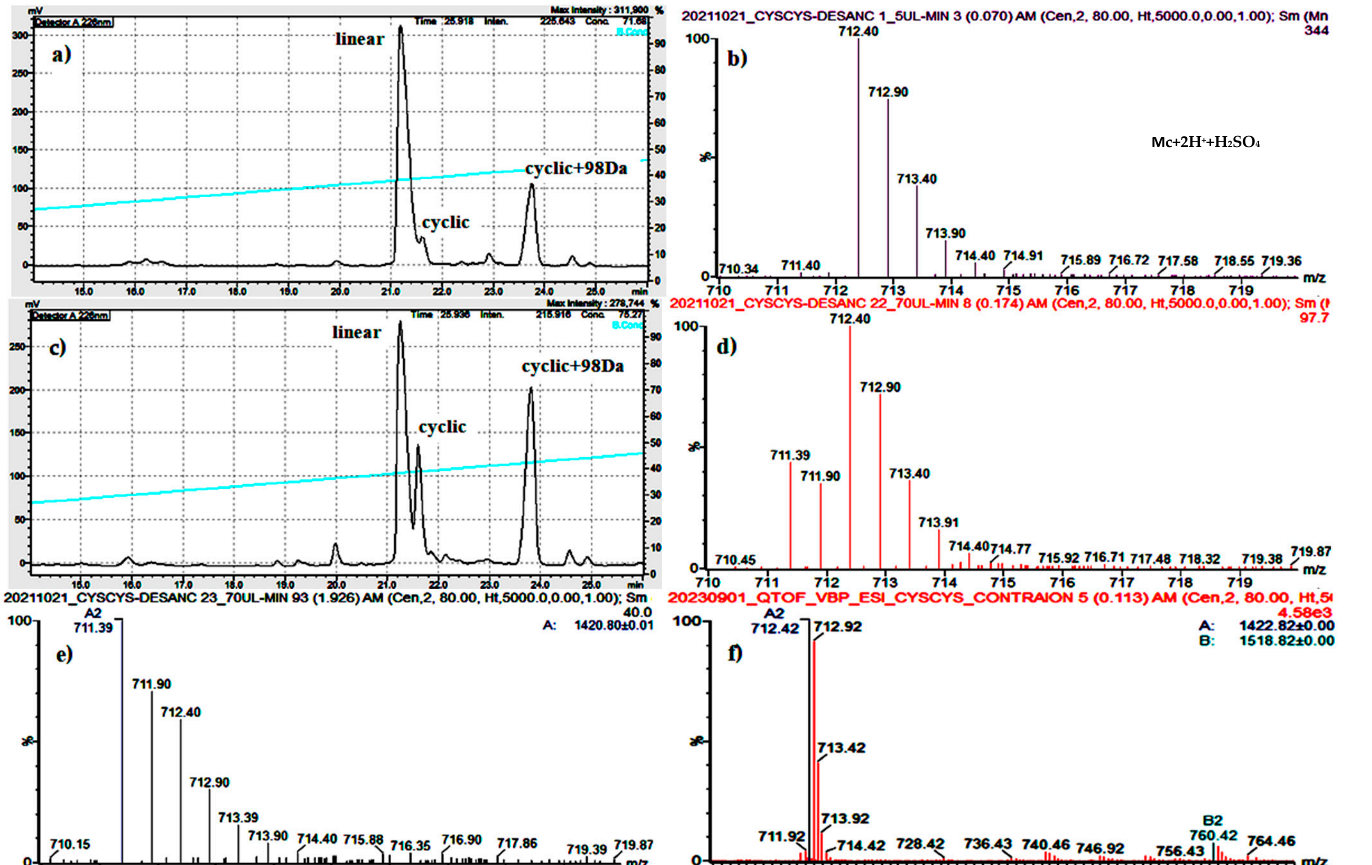

Figure S8: Analytical RP-HPLC profiles of the peptide CysCysCm-p5 after the on resin cyclization with DMSO and cleavage with 1% TIS, 3.5% H_2O (a) or 1% TIS, 1% H_2O (c). ESI-MS analysis of: peak 1 of the experiment with 3.5% H_2O (b), peak 2 of the experiment with 1% H_2O (d), peak 3 of the experiment with 1% H_2O (e), crude peptide of the experiment with 1% H_2O (not collected by RP-HPLC) (f).

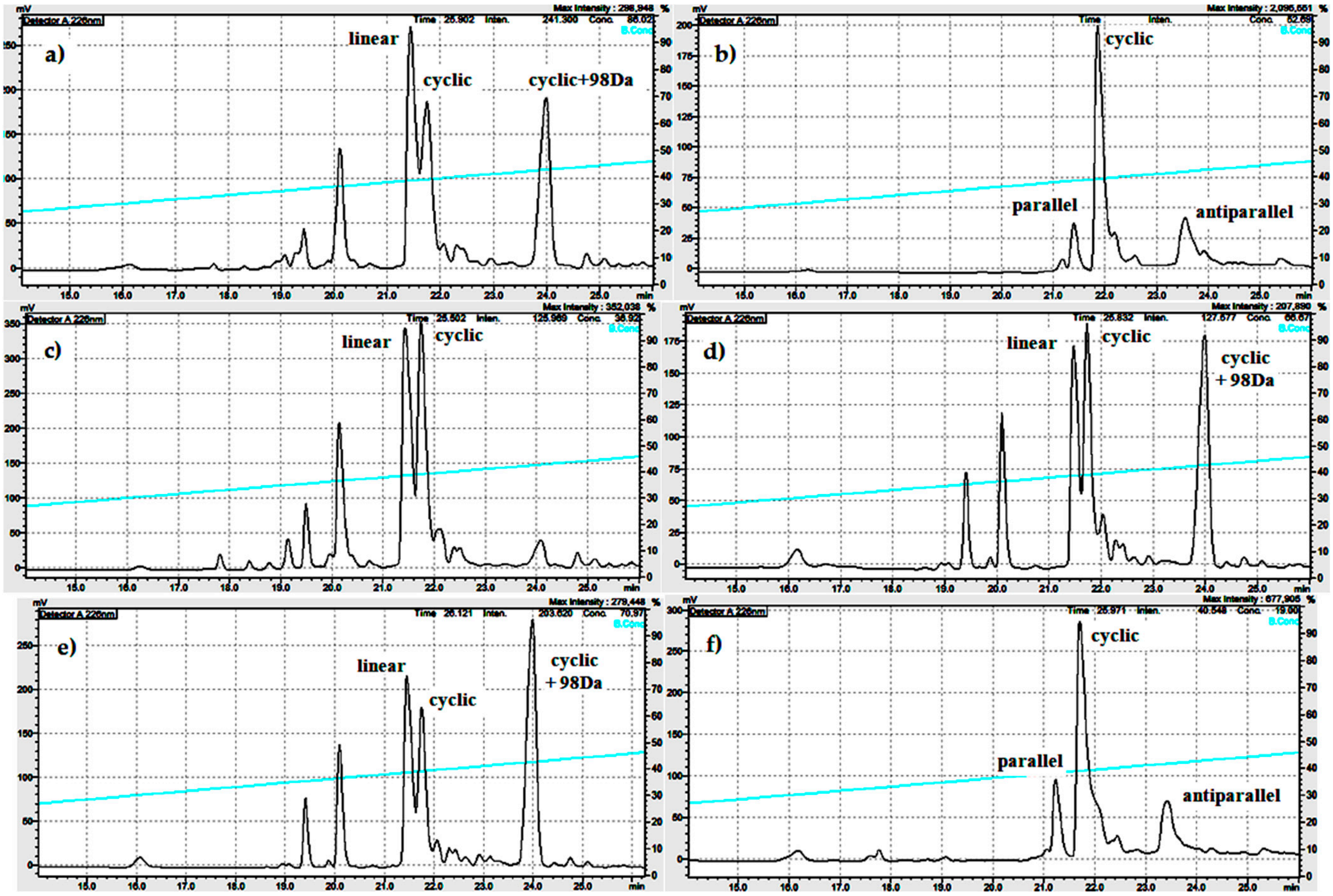

Figure S9: Analytical RP-HPLC profiles (Shimatsu HPLC system) of the crude oxidized peptide CysCysCm-p5 after the treatment with different cleavage cocktails. Chemmatrix resin of 0.2mmol/g and cleavage (1.5h) with 1% TIS (1% of $PhSiH_3$, panel c): on-resin cyclization with 35% DMSO (a, c) or I_2/DMF (b). Chemmatrix resin of 0.41mmol/g: on-resin cyclization with 35% DMSO (d), O_2/NH_3 (THF/H_2O 2:1) (e), I_2/DMF-Trt (f).

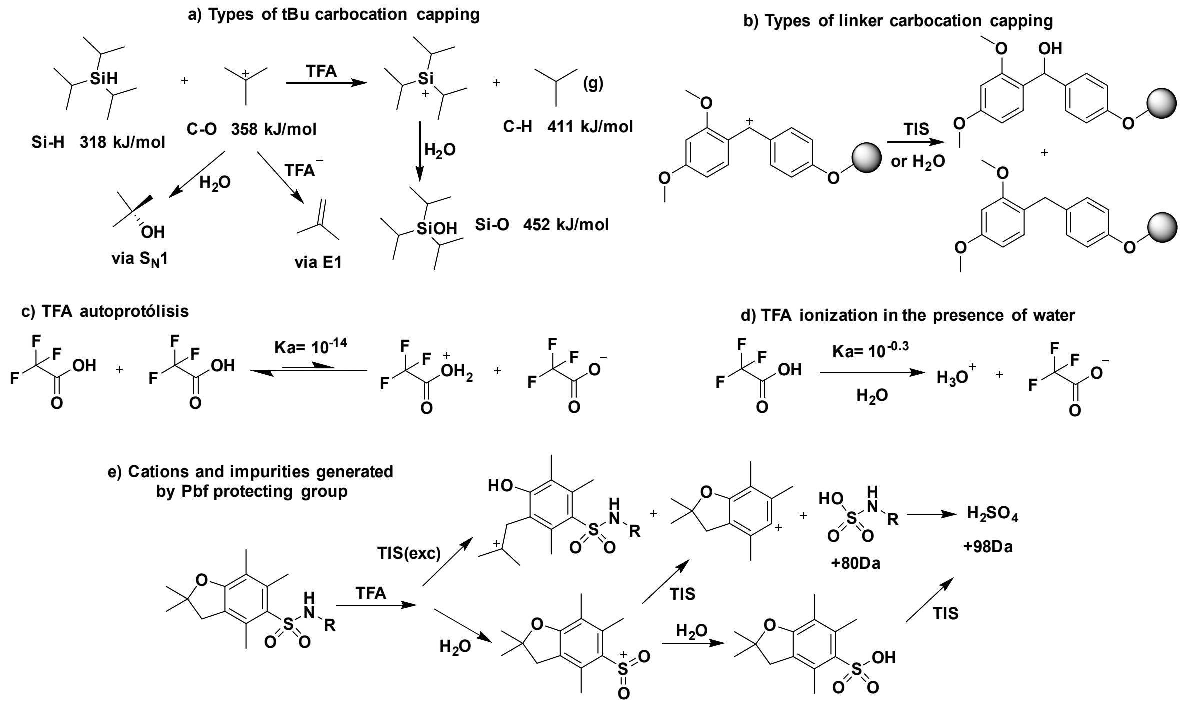

Scheme S2: (a, b) Modes of carbocation capping, (c, d) effect of water in TFA autoprotolysis and (e) impurities generated by Pbf.

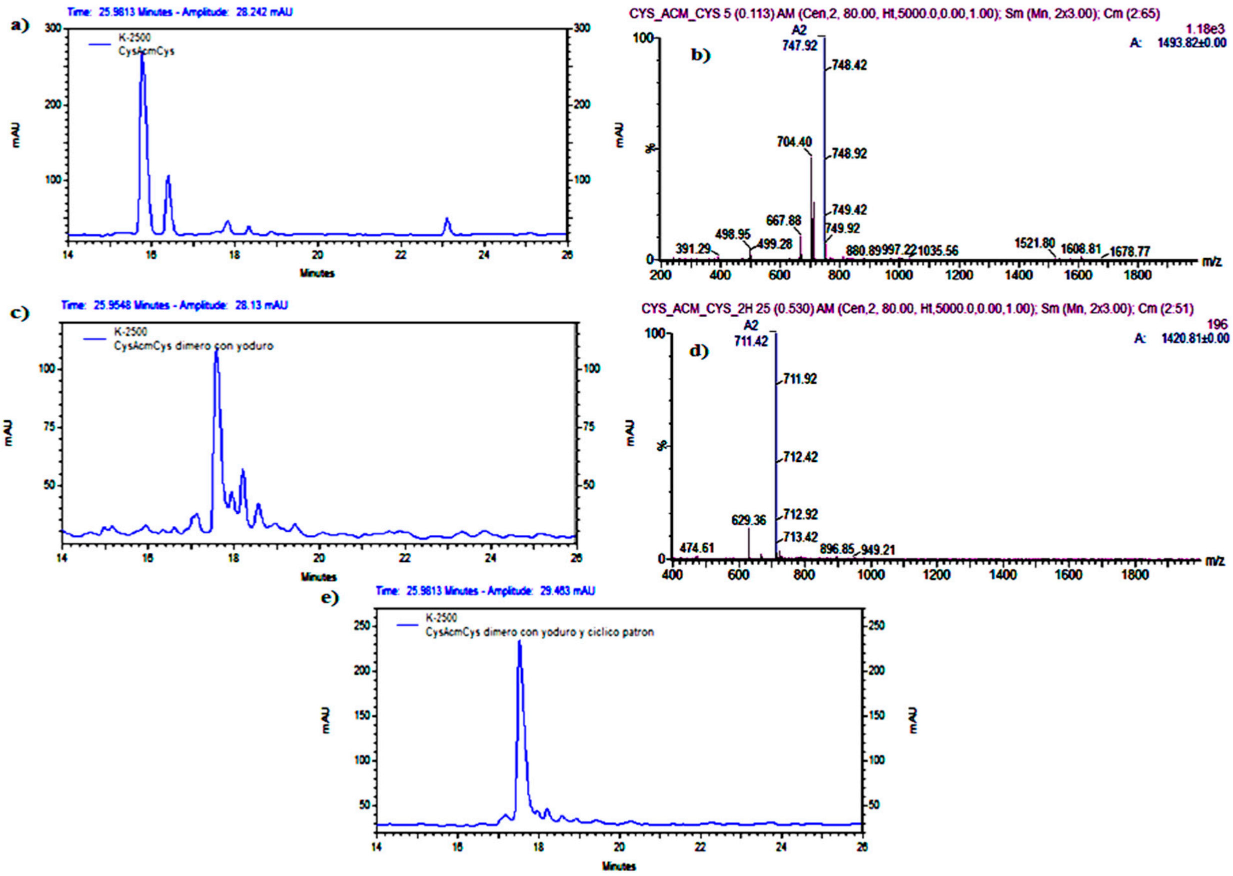

Figure S10: Analytical RP-HPLC profile (a, Knauer system) and ESI-MS (b) of crude linear peptide CysAcmCysCm-p5 and after the liquid phase cyclization (5mg/ml) with I2 in HAc (c, d) or THF (e).

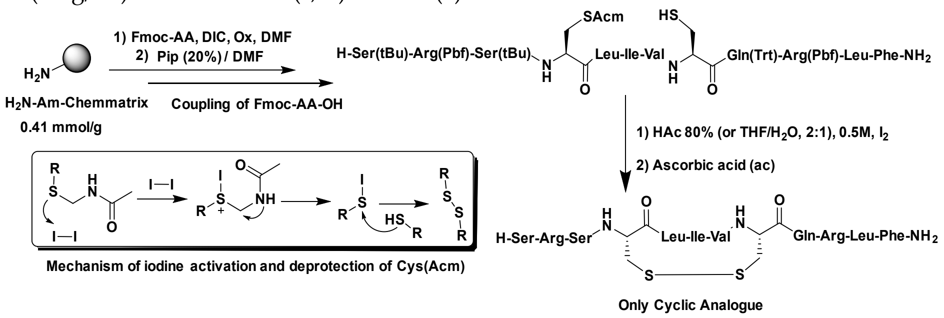

Scheme S3: Liquid phase synthesis of the cyclic CysCysCm-p5ss by iodine oxidation of the linear CysAcmCysCm-p5 peptide in HAc or THF.

2.3 *Selective synthesis of the parallel dimer of CysCysCm-p5.*

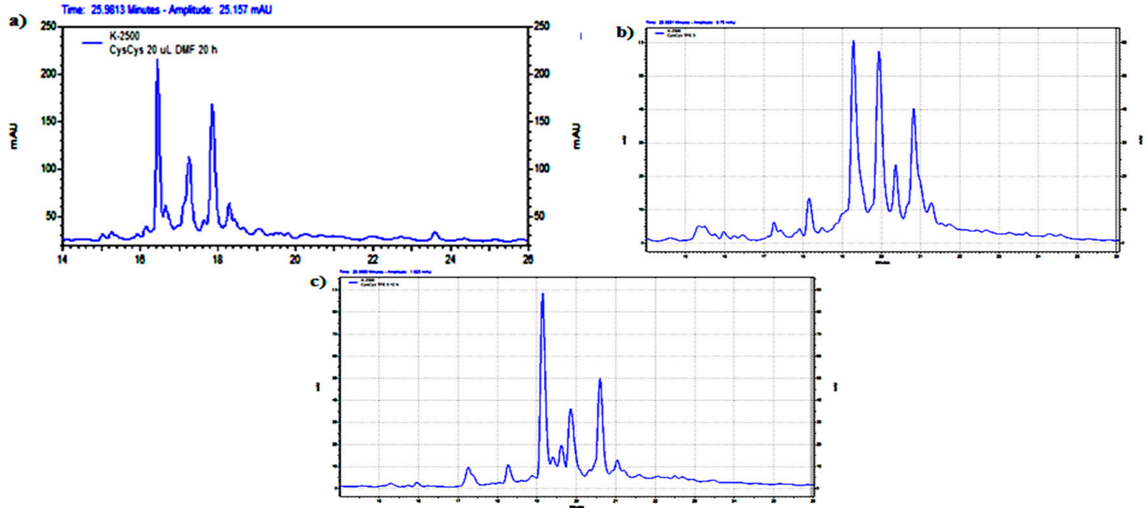

Figure S11: Analytical RP-HPLC profiles of the cyclization of CysCysCm-p5 at 0.5mg/ml in: a) 2% DMF 20h, b) 5% TFE 6h, c) 5% TFE 12h.

References

1. Annis, I.; Hargittai, B.; Barani, G. Disulfide bond formation in peptides. Methods Enzymol 1997, 289, 198-221.

2. Morales-Vicente, F.E.; González-García, M.; Díaz Pico, E.; Moreno-Castillo, E.; Garay, H.E.; Rosi, P.E.; Jimenez, A.M.; Campos-Delgado, J.A.; Rivera, D.G.; Chinea, G.; Pietro, R.C.L.R.; Stenger, S.; Spellerberg, B.; Kubiczek, D.; Bodenberger, N.; Dietz, S.; Rosenau, F.; Paixao, M.E.; Standker, L.; Otero-González, A.J. Design of a Helical-Stabilized, Cyclic, and Nontoxic Analogue of the Peptide Cm-p5 with Improved Antifungal Activity. ACS Omega 2019, 4, 19081-19095.

3. Mormann, M.; Eble, J.; Schwoppe, C.; Mesters, R.M.; Berdel, W.E.; Peter-Katalinic, J.; Pohlentz, G. Fragmentation of intra-peptide disulfide bonds of proteolytic peptides by nanoESI collision induced dissociation. Anal Bioanal Chem 2008, 392, 831-838.
